# Supplementary material for: In Situ Identification of Both IL-4 and IL-10 Cytokine–Receptor Interactions during Tissue Regeneration
Source: Cells. 2023 May 31;12(11):1522. doi: 10.3390/cells12111522 (PMC10253026; doi:10.3390/cells12111522)
Supplement: Supplementary file 1 [file cells-12-01522-s001.zip › cells-2385556-supplementary.pdf]

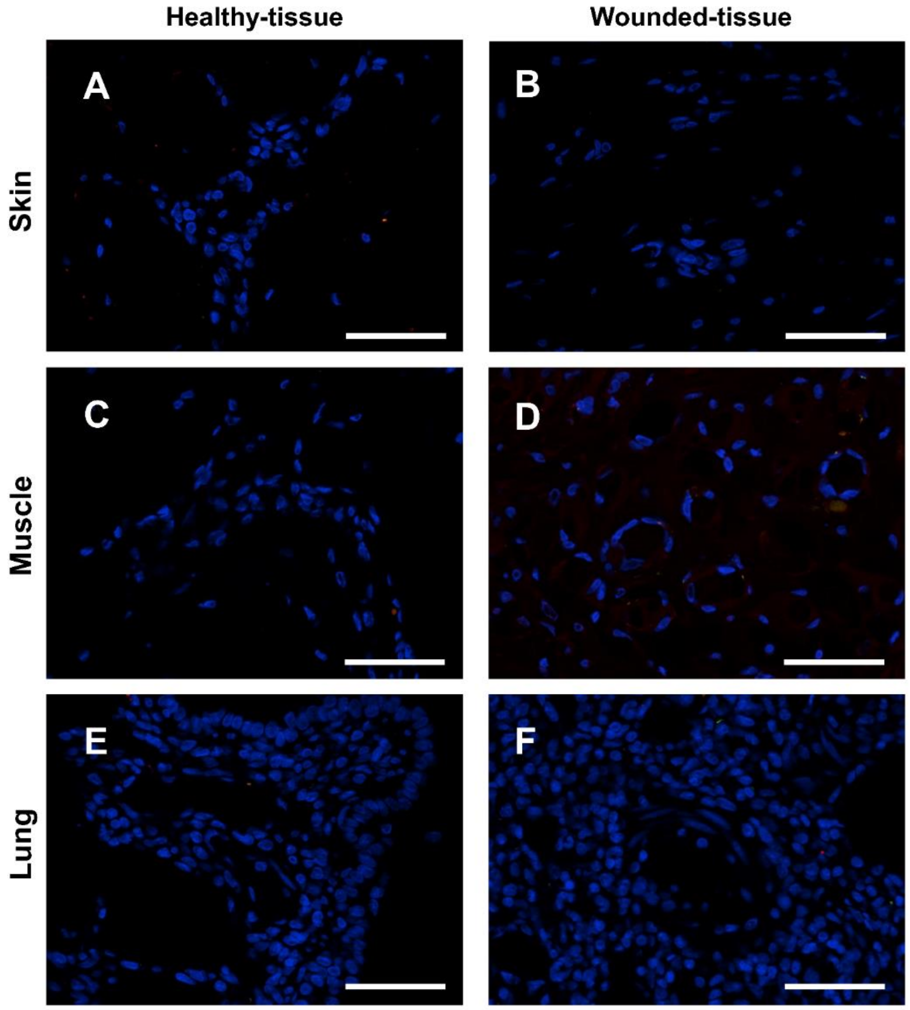

Figure S1. Figure S1. Identification of IL-4R+ and CD206+ cells in situ. (A,B) Immunolabeling with anti-IL-4R and anti-CD206 antibodies of the skin, (C,D) the muscle and (E,F) the lung sections. (A,C,E) Healthy-tissue. (B,D,F) Wounded-tissue. (A) Negative control of Figure 5A; (B) Negative control of Figure 5B; (C) Negative control of Figure 5E; (D) Negative control of Figure 5F; (E) Negative control of Figure 5I; (F) Negative control of Figure 5J,K. Anti-IL-4R (Alexa488, green fluorescence) and Anti-CD206 (Alexa568, red fluorescence) labeling the M2-like macrophages. Nuclear staining with DAPI (blue fluorescence). Scale bar = 50 µm.

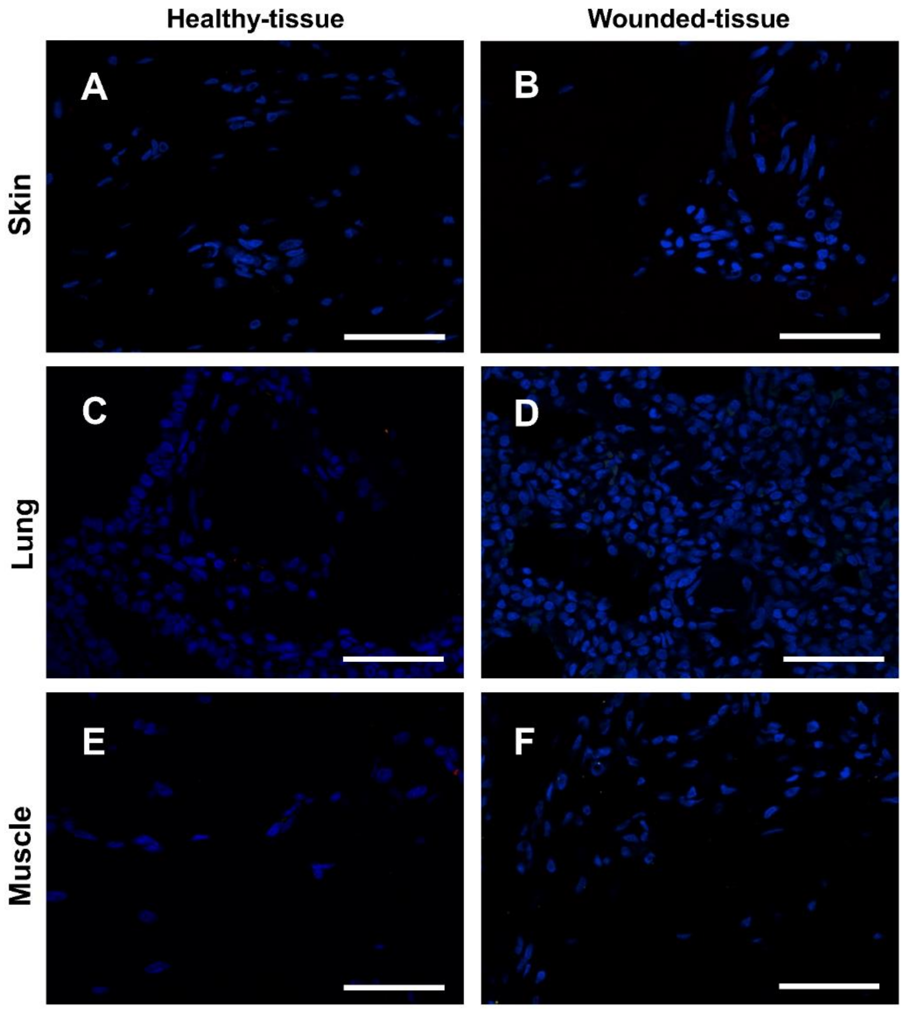

Figure S2. Identification of IL-10R+ and CD206+ cells in situ. (A,B) Immunolabeling of both anti-IL-10R and anti-CD206 antibodies of the skin, (C,D) the lung and (E,F) the muscle sections. (A,C,E) Healthy-tissue. (B,D,F) Wounded-tissue. (A) Negative control of Figure 6A; (B) Negative control of Figure 6B; (C) Negative control of Figure 6E; (D) Negative control of Figure 6F; (E) Negative control of Figure 6I; (F) Negative control of Figure 6J-L. Anti-IL-10R (Alexa488, green fluorescence) and Anti-CD206 (Alexa568, red fluorescence) labeling the tissue-resident and M2-like macrophages. Nuclear staining with DAPI (blue fluorescence). Scale bar = 50 μm.

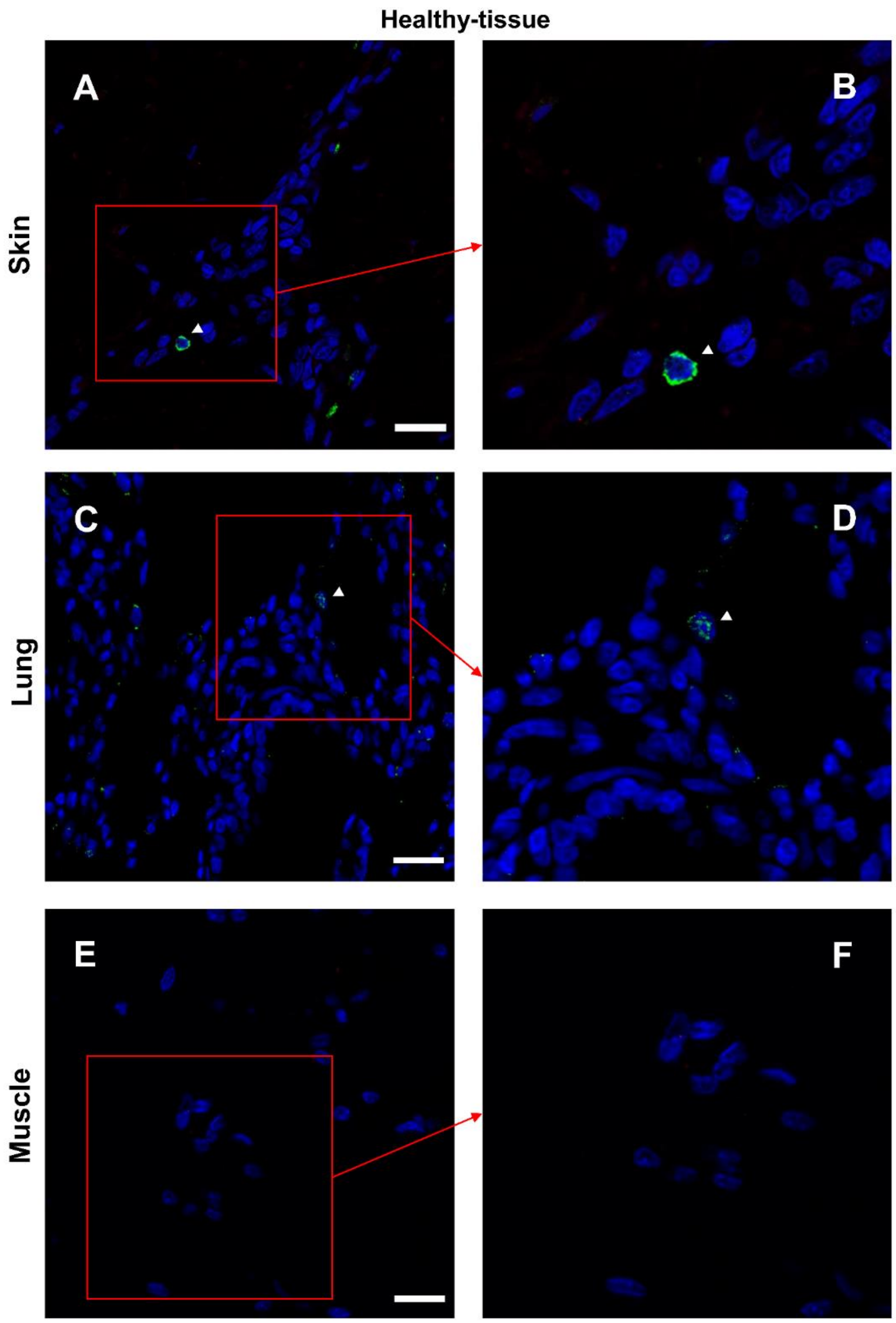

Figure S3. Identification of IL-4R+ and IL-4+ cells in situ. (A,B) Immunolabeling of both anti-IL-4R and IL-4 antibodies in the healthy skin, (C,D) the lung and (E,F) the muscle sections. (B,D,F) Expended view: high magnification image of the area within the red rectangle in A,C,E. Anti-IL-4R (Alexa488, green fluorescence) and Anti-IL-4 (Alexa568, red fluorescence). Nuclear staining with DAPI (blue fluorescence). Scale bar = 20 μm. Arrowhead: IL-4R+ cells.

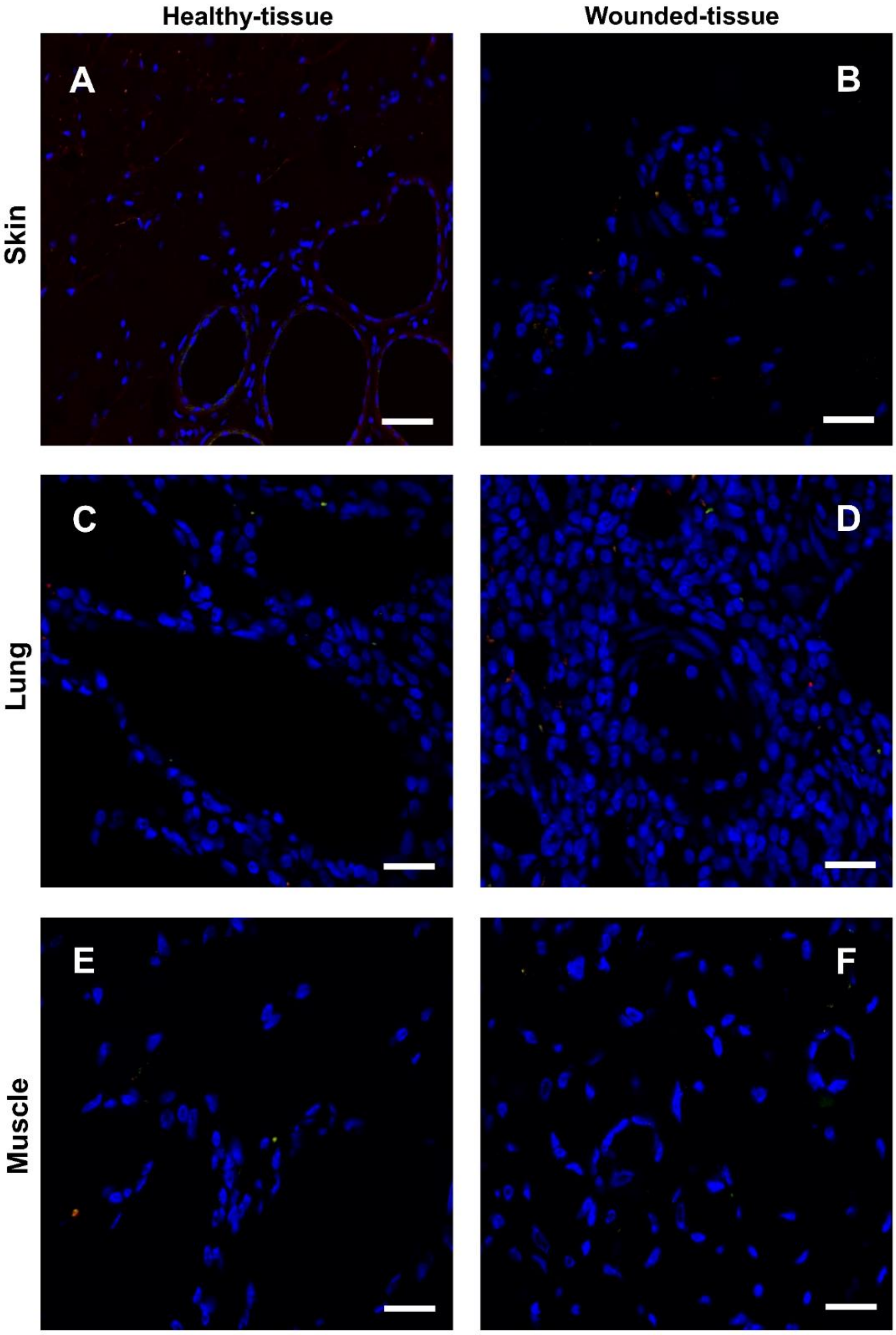

Figure S4. Identification of IL-4R+ and IL-4+ cells in situ. (A,B) Immunolabeling of both anti-IL-4R and IL-4 antibodies in the skin, (C,D) the lung and (E,F) the muscle sections. (A,C,E) Healthy-tissue. (B,D,F) Wounded-tissue. (A) Negative control of Figure S3A,B; (B) Negative control of Figure 7A,B; (C) Negative control of Figure S3C,D; (D) Negative control of Figure 7C,D; (E) Negative control of Figure S3E,F; (F) Negative control of Figure 7E,F. Anti-IL-4R (Alexa488, green fluorescence) and Anti-IL-4 (Alexa568, red fluorescence). Nuclear staining with DAPI (blue fluorescence). Scale bar = 20 μm.

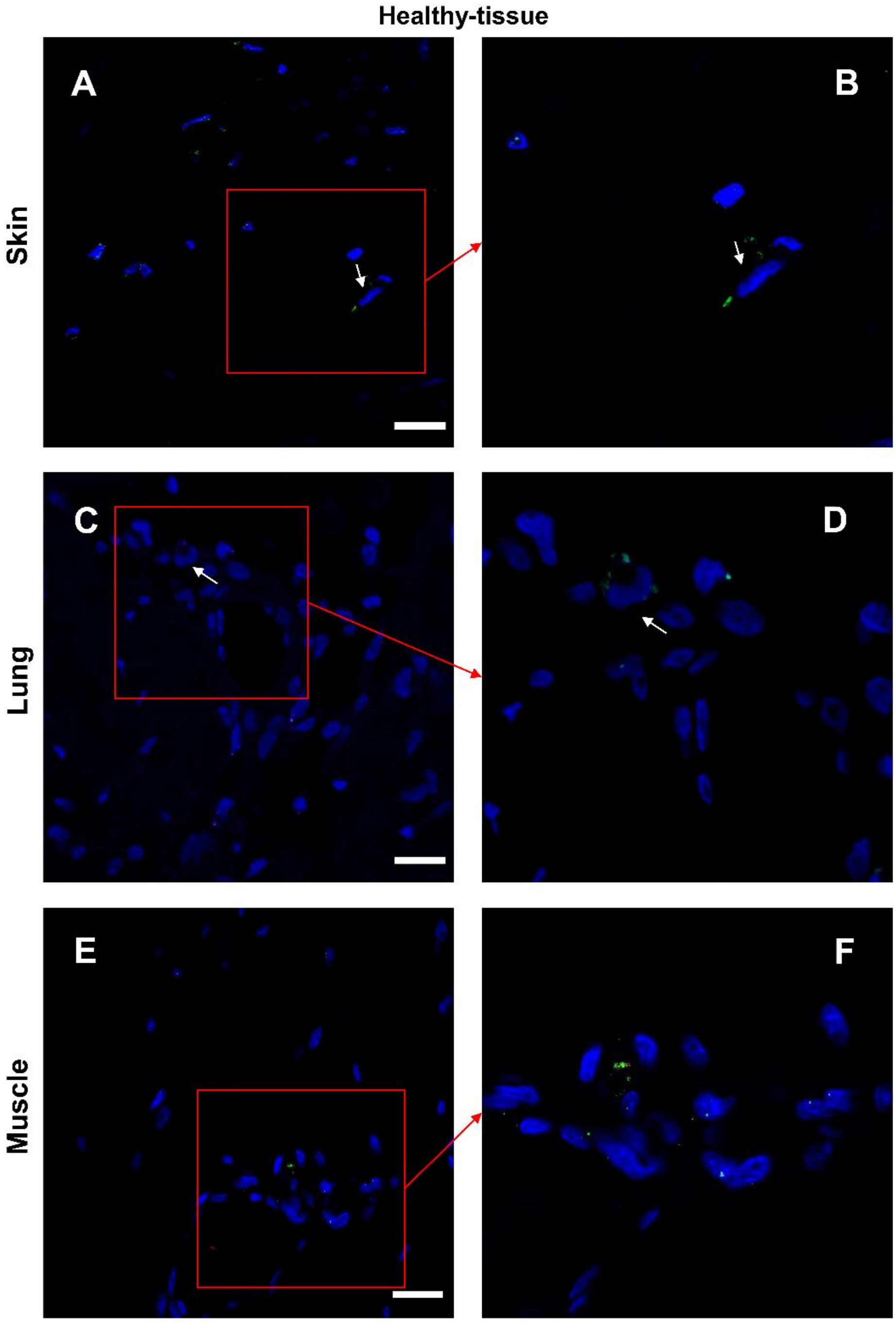

Figure S5. Identification of IL-10R+ and IL-10+ cells in situ. (A,B) Immunolabeling of both anti-IL-10R and IL-10 antibodies in the healthy skin, (C,D) the lung and (E,F) the muscle sections. (B,D,F) Expanded view: high magnification image of the area within the red rectangle in A,C,E. Anti-IL-10R (Alexa488, green fluorescence) and Anti-IL-10 (Alexa568, red fluorescence). Nuclear staining with DAPI (blue fluorescence). Scale bar = 20 μm. Thin arrow: IL-10R+ cell.

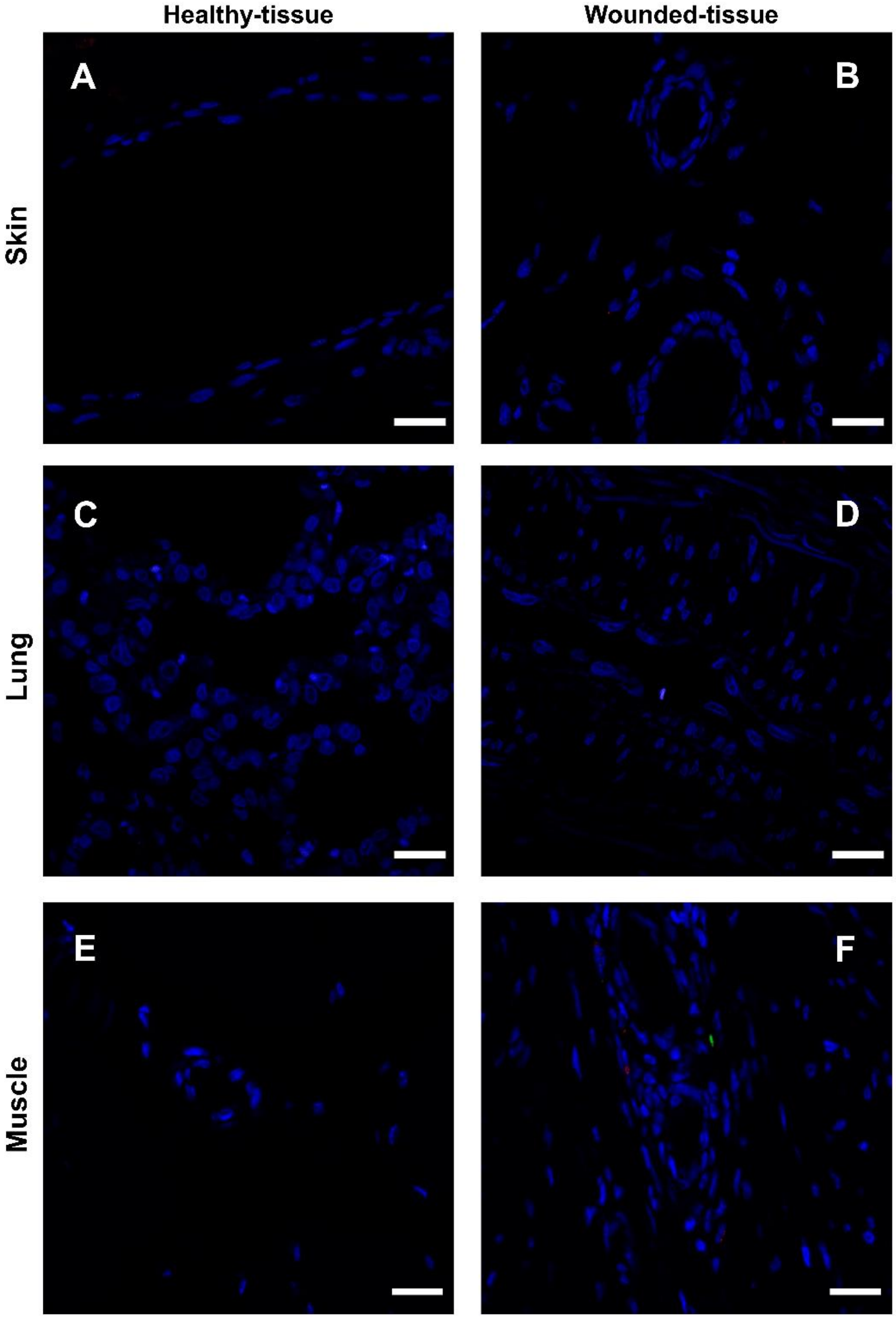

Figure S6. Identification of IL-10R+ and IL-10+ cells in situ. (A,B) Immunolabeling with anti-IL-10R and IL-10 antibodies in the skin, (C,D) the lung and (E,F) the muscle sections. (A,C,E) Healthy-tissue. (B,D,F) Wounded-tissue. (A) Negative control of Figure S5A,B; (B) Negative control of Figure 8A,B; (C) Negative control of Figure S5C,D; (D) Negative control of Figure 8C,D; (E) Negative control of Figure S5E,F; (F) Negative control of Figure 8E,F. Anti-IL-10R (Alexa488, green fluorescence) and Anti-IL-10 (Alexa568, red fluorescence). Nuclear staining with DAPI (blue fluorescence). Scale bar = 20 μm.

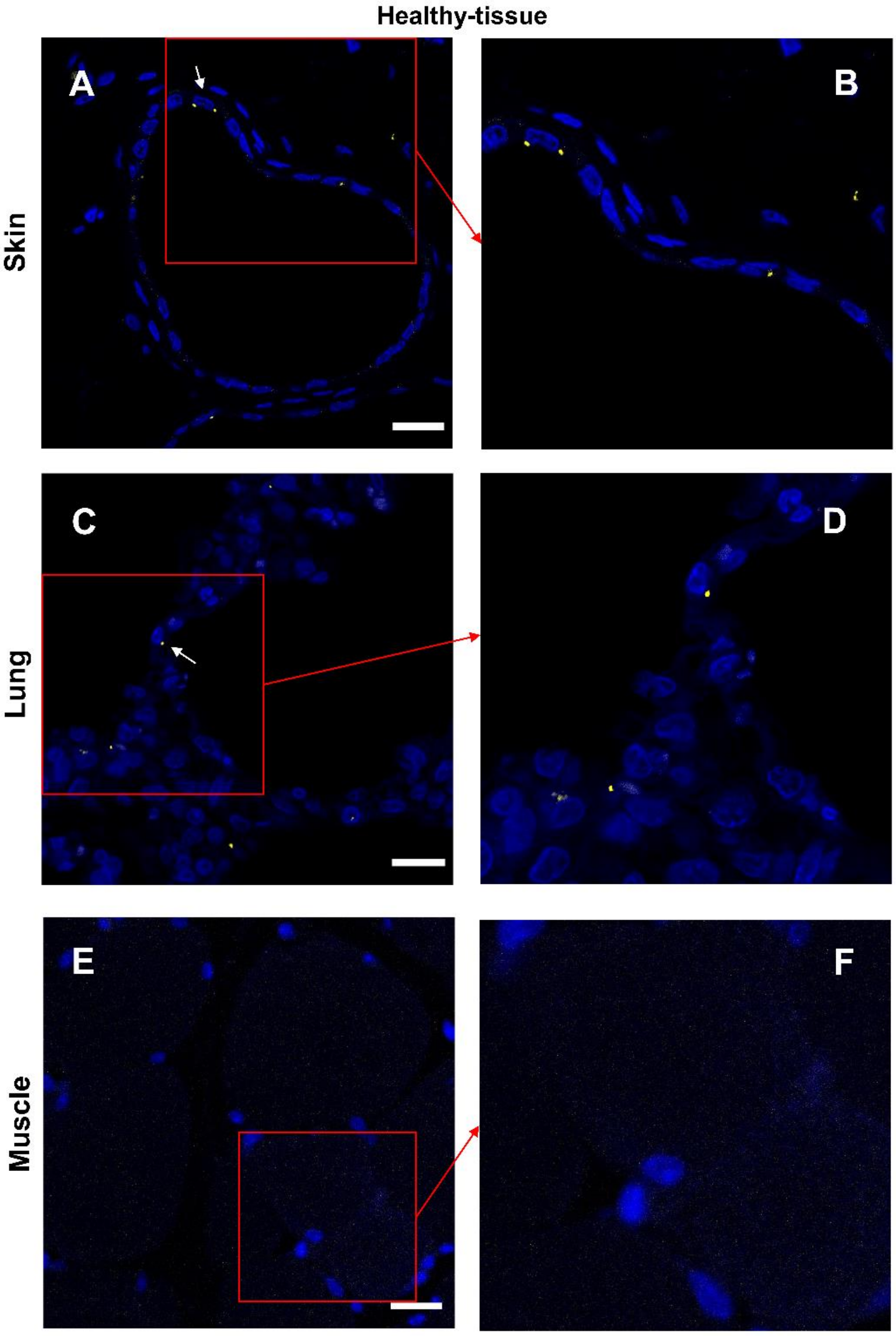

Figure S7. Interaction between IL-4 and IL-4 IL-4R in situ. (A,B) Proximity Ligation Assay with anti-IL-4R and anti-IL-4 antibodies in the healthy skin, (C,D) the healthy lung and (E,F) the healthy muscle. (B,D,F) Expanded view: high magnification image of the area within the red rectangle in A,C,E. Alexa568, yellow fluorescence, labeling the interaction between IL-4 and IL-4R. Nuclear staining with DAPI (blue fluorescence). Scale bar = 20 µm. Arrow: Proximity Ligation Assay IL-4R/IL-4 positive cells.

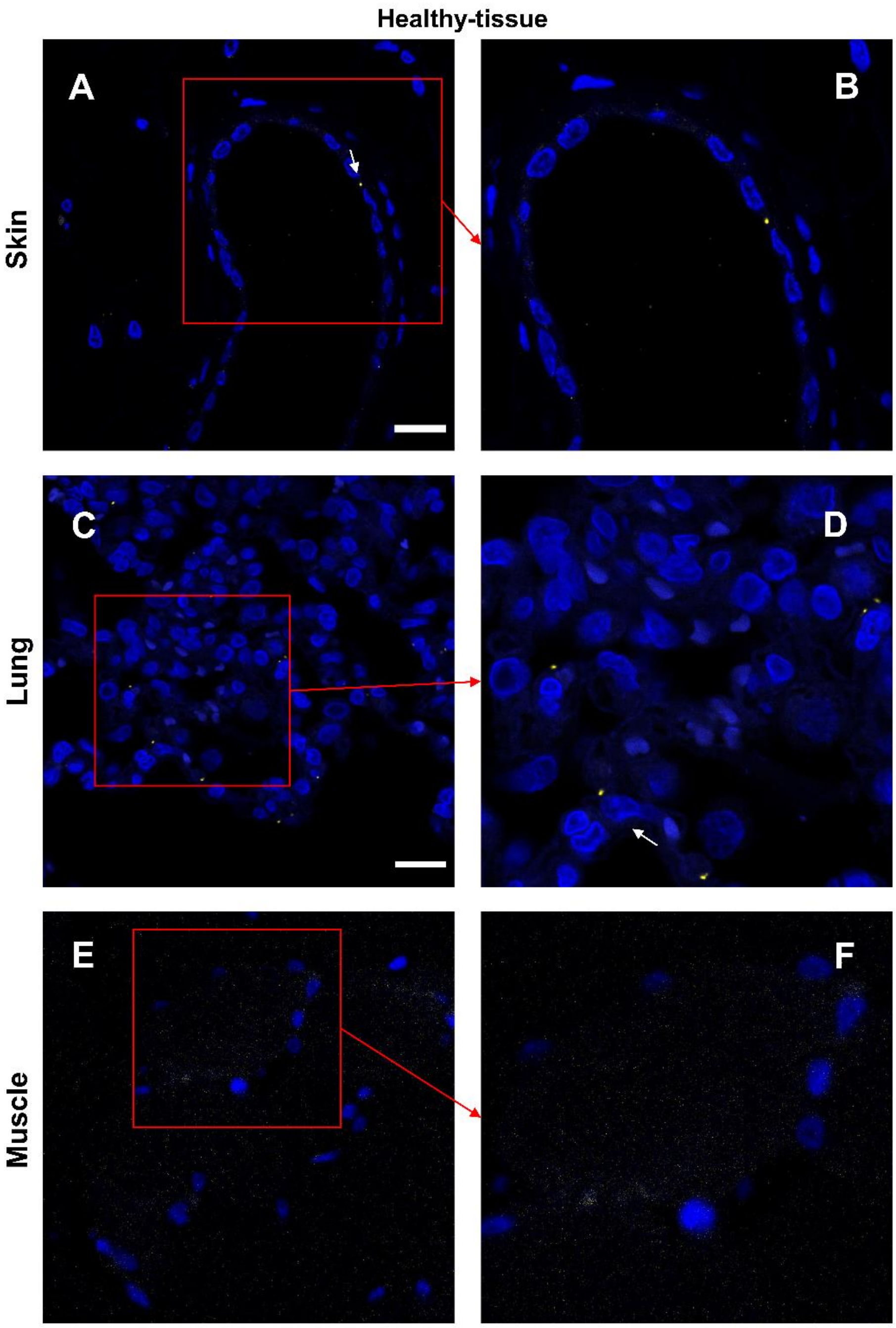

Figure S8. Interaction between IL-10 and IL-10R in situ. (A,B) Proximity Ligation Assay with anti-IL-10R and anti-IL-10 in the healthy skin, (C,D) the healthy lung and (E,F) the healthy muscle. (B,D,F) Expanded view: high magnification image of the area within the red rectangle in A,C,E. Alexa568, yellow fluorescence, labeling the interaction between IL-10 and IL-10R. Nuclear staining with DAPI (blue fluorescence). Scale bar = 20 μm. Arrow: Proximity Ligation Assay IL-10R+/IL-10+ cells.

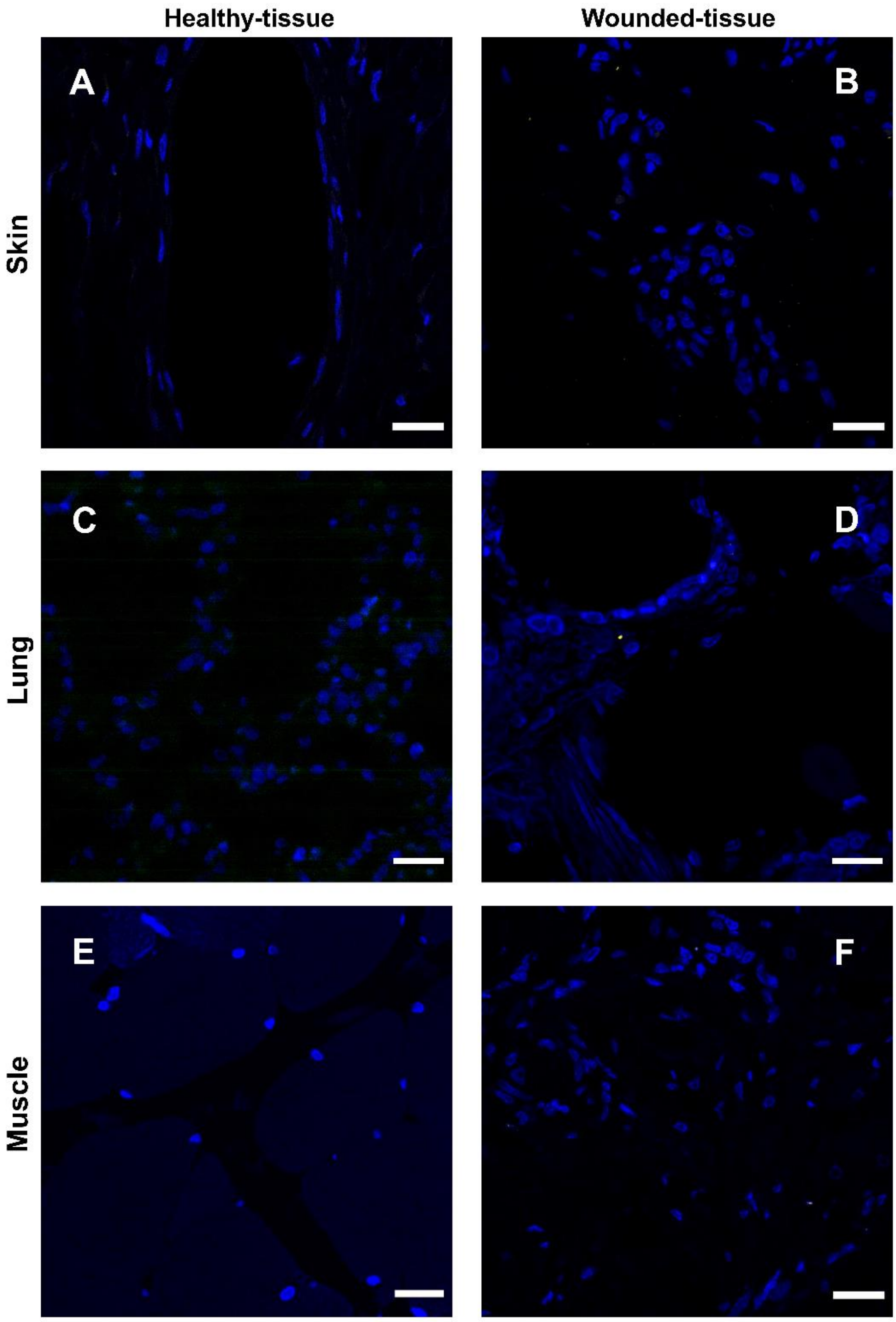

Figure S9. Interaction between IL-4/IL-4R and IL-10/IL-10R in situ. (A,B) Proximity Ligation Assay with anti-IL-4 and anti-IL-4R; anti-IL-10 and anti-IL-10R antibodies in the skin, (C,D) the lung and (E,F) the muscle sections. (A,C,E) Healthy-tissue. (B,D,F) Wounded-tissue. (A) Negative control of Figure S7A,B and S8A,B; (B) Negative control of Figure 9A,B and 10A,B; (C) Negative control of Figure S7C,D and S8C,D; (D) Negative control of Figure 9C,D and 10C,D; (E) Negative control of Figure S7E,F and S8E,F; (F) Negative control of Figure 9E,F and 10E,F. Alexa568, yellow fluorescence, labeling the interaction between IL-4 and IL-4R; IL-10 and IL-10R. Nuclear staining with DAPI (blue fluorescence). Scale bar = 20 μm.
